# Effects of a 6-Month Exercise Intervention on Primitive Reflexes in Children with Developmental Language Disorder—A Case for Multisensory and Sensorimotor Integration

**DOI:** 10.3390/children12121616

**Published:** 2025-11-27

**Authors:** Brigitta Tele-Heri, Krisztina Csapo, Janos Szabo, Csaba Papp, Rudolf Gesztelyi, Judit Zsuga

**Affiliations:** 1 Department of Psychiatry, Faculty of Medicine, University of Debrecen, Nagyerdei krt. 98, 4032 Debrecen, Hungary; tele-heri.brigitta@sph.unideb.hu (B.T.-H.); csapo.krisztina@med.unideb.hu (K.C.); szaboj@med.unideb.hu (J.S.); 2 Kálmán Laki Doctoral School, University of Debrecen, Nagyerdei krt. 98, 4032 Debrecen, Hungary; 3 Institute for Primary Care and Health Promotion of Debrecen, Clinical Center, University of Debrecen, Nagyerdei krt. 98, 4032 Debrecen, Hungary; papp.csaba@med.unideb.hu; 4 Department of Pharmacology and Pharmacotherapy, Faculty of Medicine, University of Debrecen, Nagyerdei krt. 98, 4032 Debrecen, Hungary; gesztelyi.rudolf@pharm.unideb.hu

**Keywords:** multisensory integration, sensorimotor integration, individualized vestibular exercises, primitive survival reflexes, receptive language performance

## Abstract

**Objectives**: Language is one of the core attributes of human development. Impaired or delayed language development (i.e., developmental language disorder: DLD) is a highly prevalent condition; however, its underlying etiopathogenetic causes are not fully elucidated. The possible role of multisensory integration (MSI) may be proposed. The aim of this pilot interventional study was to assess the effect of an individualized vestibular exercise training program regarding the processes that rely on multisensory integration in DLD. **Methods**: Children aged between 5 and 12 years with DLD and their age-matched neurotypical controls were included. Following informed consent, a baseline assessment (primitive reflexes, postural control, receptive language performance) was conducted. Next, a 26 week-long exercise program rich in vestibular stimuli was implemented in the DLD group. At 26-week follow-up, both groups were reassessed. **Results**: Compared to baseline, the primitive reflex profile significantly improved in the DLD group. Scores for dynamic postural control also improved (score of 0.25 IQR 0–1 at baseline vs. 2 IQR 1–2 at follow-up; *p* < 0.001). Age-standardized scores for receptive grammar (score of 79.5 IQR 71.5–89.5 at baseline, 87 IQR 66–103 at follow-up; *p* = 0.03) were also improved. When two-way comparisons using the mixed-effects models were made, improvement in the DLD group was evident when compared to baseline levels and to the control group at follow-up. **Conclusions**: Based on these results, the possible interplay between multisensory and sensorimotor integration and integration of primitive reflexes is proposed, with vestibular stimulation contributing to the cortical input that may underlie the maturation of the areas dedicated to multisensory processes.

## 1. Introduction

Language is one of the core attributes of human development, one that profoundly impacts learning ability and social functioning [1, 2, 3]. Impaired or delayed language development is a highly prevalent condition, impacting around 7% of children; however, its underlying etiopathogenetic causes are not fully elucidated [4]. The recent consensus-based definition of developmental language disorder (DLD) delineates language disorders and DLD based on the presence or absence of differentiating conditions (e.g., brain injury, acquired epileptic aphasia, cerebral palsy, genetic causes, autism spectrum disorder, etc.), respectively. This distinction carries implications regarding the etiology, prognosis, and efficacy of interventions; hence, it is proposed that DLD is multifactorial in nature and shows a highly heterogenous presentation [2]. Accordingly, DLD is defined as a “persistent language disorder of unknown etiology having functional impact on everyday social interactions or learning” [4], a disorder that emerges in the context of development in lieu of being acquired due to a biomedical cause [2]. Nevertheless, the aim to identify the causal developmental factors contributing to the evolution of DLD is ongoing.

A possible common factor underlying DLD may be the disturbance of multisensory integration (MSI). Increasing empirical findings suggest that multisensory integration scaffolds the development of language, cognition, and higher-order skills [5]. Impaired multisensory integration has been proposed to have a cascading effect on language development; in fact, the facilitation of multisensory function has been proposed as an important target for interventions [6]. MSI is a mechanism that has evolved to cope with complex environments immersed in sensory signals of varying intensity by means of dedicated multisensory (MS) neurons. These MS neurons identify and respond to cross-modal attributes such as rhythm, temporal synchrony, and common spectral information [7, 8]; hence, a single MS neuron combines the corresponding sensory information yielded by the cross-modal attributes of sensory signals, and thus MSI is more powerful if cross-modal inputs are aligned to the expected interrelation of senses [9]. The maturation of MSI shows a protracted course during postnatal life [7, 9, 10], showing vulnerability to developmental insults. It has developmental implications, as it plays an important role in postural stability, e.g., providing sustained control of the multi-segmental body by means of MSI and related sensorimotor integration (SMI) [11, 12]. Initially, young infants rely mainly on vision, as the ability to reweight visual and touch sensory information emerges at around 4 years of age [13]. It is between the ages of 9–12 years that all three sensory modalities (including vestibular cues) feed into the multisensory inputs for postural control [10, 14].

The role of MSI in language development has been proposed previously [15]. Sensitivity to the receptive attributes of speech, e.g., rhythmic and prosodic cues, has been suggested to be an antecedent of language acquisition [16]. Conversely, phonological deficits in DLD were shown to be multisensory in nature [17]. Furthermore, MSI was shown to assume a role in language development by detecting temporal correlation and rhythmic regularities between auditory input and orofacial movement [18, 19]. Impaired MSI with respect to visual and auditory attributes of spoken language has been investigated in the context of DLD. In a study with 20 children with DLD and their age-matched typically developing (TD) peers, audiovisual speech synchrony was assessed. The authors found that, in addition to speech perception, the ability to process temporal coherence between auditory and visual attributes of spoken language was impaired in children with DLD as opposed to their TD peers. Furthermore, children with DLD had difficulty in distinguishing between temporally coherent and incoherent auditory and visual attributes of speech [20]. Similarly, among a group of children with a history of DLD, an event related potential (ERP) study reported impaired audiovisual temporal discrimination, possibly due to the reduced sensitivity to temporal asynchrony of audiovisual input [21].

Maturational delay corresponding to functional neurological disorders may be indicated by retained primitive reflexes. In fact, retained primitive reflexes were recently proposed as potential biomarkers for developmental disabilities [22]. Primitive reflexes are transiently present at birth and are instrumental for postnatal survival. They are automatic movement patterns elicited by labyrinth-induced vestibular input [23] (for a detailed overview, see Appendix A) [24, 25, 26, 27]. Nevertheless, early presence, followed by programmed inhibition, is mandatory for the timely development of numerous higher-order processes [28]. These include posture, balance, and coordination, which are perceptual processes underlying language, reading, and writing skill development [23, 28]. During typical development, primitive reflexes are inhibited by the stereotyped repetition of reflex movements [29]. If retained beyond 6–12 months of age, they hinder sensory processing, MSI, and related sensorimotor integration [27, 30]. Previously, retained primitive reflexes were reported among young children with DLD. The authors suggested that there is a relationship between language development and the course of primitive reflex integration [31]. Furthermore, it was proposed that movement programs that facilitate the integration of retained primitive reflexes may be beneficial for improving language skills. Further empirical evidence supports this research avenue. A 12-week rhythm-, balance-, and coordination-focused motor program was shown to facilitate the integration of specific primitive reflexes and contribute to behavioral improvements in children with neurodevelopmental disorders [32]. Similarly, a classroom-based exercise program was shown to reduce or fully inhibit primitive reflexes parallel to improving communication skills in developmental language disorder [33].

The current pilot study is based on the hypothesis that vestibular stimulation, by strengthening MSI and related sensorimotor integration, facilitates the integration of retained primitive reflexes, postural control, and language development [24]. Therefore, an individualized exercise program assessing the specific influence of vestibular stimulation (individualized vestibular exercise (IVE) program) was delivered to children with developmental language disorder. Changes in primitive reflex profile, postural control, and receptive language performance were assessed, given their reliance on multisensory and sensorimotor integration.

## 2. Materials and Methods

### 2.1. Study Population and Study Design

This is a pilot interventional study—a small, non-randomized pre–post study with a passive control group and about 10% dropout—to assess the specific influence of vestibular stimulation. As participants were minors, they were informed about the study and were asked for their consent to participate. At the same time, their parent(s) gave their written informed consent for the participation of their child. Participants in the DLD group were prospectively recruited between 6 September 2021 and 26 November 2021. The inclusion criteria were DLD, age between 5 and 12 years, ability to cooperate with the physiotherapist, and willingness of parents to execute the 26-week-long IVE program. To distinguish between DLD and language disorders associated with a biomedical condition, exclusion criteria consisted of differentiating conditions [2] (presence of cerebral palsy or tumor in case history, known heritable disease contributing to atypical language development (e.g., Down syndrome, autism spectrum disorder, etc.)). Following informed consent from the parents and the children, the baseline assessment was conducted. This was followed by the 26-week IVE program (for basic principles, see [24]). The follow-up period was 26 weeks long to allow for the emergence of the chronic effects of the exercise program [34]. Upon completion, the children were reassessed. Regarding the control group, age-matched children with typical development (TD) were invited to participate. Children in the control group underwent the same investigations at baseline and at 26 weeks to account for general developmental changes. Due to lack of parental commitment to engage in delivering a general regular exercise program during the 26-week-long follow-up period, children in the control group received no active treatment; hence, this was a passive control group. Participants were recruited between 6 September 2021 and 30 November 2022. The present study was approved by the Ethical Committee of the University of Debrecen (DE RKEB/IKEB 5791/2021); the investigation conforms to the principles outlined in the Declaration of Helsinki.

Detailed case history (regarding the pregnancy of the mother, perinatal events, rehabilitation history and use of current medication) was obtained. Every participant was medication-naive. Information from multiple sources were combined when establishing the presence of developmental language disorder. Parents were asked about their own observations and the reflections of the adults taking care of their child regarding signs indicative of atypical language development (difficulty with producing a narrative, understanding spoken language, impaired ability to follow oral instructions, having difficulties engaging in reciprocal conversation) [1] or whether their child has been diagnosed previously as having DLD. Children were included in the DLD group if the parental/caretaker reports indicated signs of atypical language development and/or a formalized speech–language assessment indicated lower performance in the domains of language and emergent literacy skills using the logopedic test Szól-e [35].

Neurodevelopmental examination was performed by assessing five main domains: central nervous system maturity, gross motor skills, body schema perception, tactile perception, and rhythm [36]. The persistence of primitive survival reflexes and postural development was assessed by the physiotherapist investigator.

The primary endpoint was the composite of the primitive reflex profile score. This was compiled to characterize the overall primitive reflex profile (ranging from 0 to 8 points) in the mixed-effects model and linear regression. The components of the composite score included scores for the primitive survival reflexes—TLR (tonic labyrinth reflex), ATNR (asymmetric tonic neck reflex), and STNR (symmetric tonic neck reflex)—and the Schilder test, all assessed as described previously [36]. Shortly, to assess the presence of TLR, three tasks were performed, each scored on a scale of 0–2, denoting the absence of TLR. The mean of these three scores was used as input for the summary primitive reflex profile score. First, the children were asked to lay prone on a skateboard holding their arms and legs lifted upwards, with palms facing each other, for 12 s. Scoring was between 2 and 0 points, with 2 points given for holding the position for 12 s and 0 points for lowering extremities within 10 s. Next, execution of a forward summersault was assessed starting from a squatting position with palms on the floor facing ahead (from 2 to 0 points). Finally, the children were asked to lay supine, holding their arms, palms facing each other, feet and head elevated for 12 s (2 points for keeping the position for minimum 12 s, 0 points for stopping before 10 s). The sum of the scores for the three probes was used for statistical analysis. To assess the presence of STNR, children started out from a hand-and-knees position with the head looking ahead. Next, the children were instructed to look downward slowly, over 4 s, in a way such that their chin reached their chest, and then, slowly, over the course of 4 s, to look up to the ceiling and repeat this maneuver. We asked the children to repeat this maneuver 5 times. Scores between 2 and 0 were given for the absence or presence of STNR, respectively. Asymmetric tonic neck reflex was assessed by having the children get in a hands-and-knees position and asking them to look straight ahead, and then, slowly, over 4 s, to turn their head to the left and then, over 4 s, to the right. We asked the children to repeat this maneuver 5 times. Scores between 2 and 0 were given for the absence or presence of ATNR, respectively. ATNR was further assessed by performing the Schilder test (scored 2 for the lack of and 0 for the presence of ATNR). Secondary outcome measures included postural stability and a measure of receptive language, both of which processes are rooted in multisensory processes. In order to assess dynamic postural stability, the children were asked to sit cross-legged on a skateboard, holding the examiner’s hand (with their arms and trunk being perpendicular). The children were instructed to maintain their posture and their arms extended while the examiners pulled or pushed them for 10–12 feet. Scores between 2 and 0 were given for the absence or presence of co-contraction, respectively, when being pulled and pushed, and the arithmetic mean of the two scores was used for analysis. Receptive language performance was assessed by administering the Test for Reception of Grammar-2. The results are presented as the age-normalized standardized points reflective of the Hungarian population, as provided by the Hungarian handbook of TROG-2 [37]. The reason for opting to assess receptive language performance and not expressive language comes from the trajectory of language development. It is well established that early insight into the child’s receptive language development is paramount in terms of identifying the need and providing the means to support communication with the child. As early caregiver intervention may facilitate language and communication development, the identification of delays in receptive language development is clinically important [38].

A speech and language therapist (holding a bachelor of arts degree in special needs education and speech and language therapy) conducted the baseline logopedic evaluation using the logopedic test Szól-e [35]. Every other examination (e.g., taking the case history, the TROG-2 test, assessment of neurodevelopment, primitive survival reflexes, and postural stability) was conducted by the same physiotherapist (referred to as the investigator) holding a bachelor’s degree in physiotherapy, a master’s degree in complex rehabilitation, and specialized training in diagnosing neurodevelopmental delays and sensorimotor therapy (hence, inter-rater variability was not an issue). The investigator was not blinded.

For the treatment group, an individualized exercise plan was administered for every child in a supervised manner. Each exercise plan consisted of active and passive vestibular exercises. The IVE program builds on some basic principles. Its foundation is the utilization of the vestibular stimuli to drive neurodevelopment. Additionally, it exploits rhythm, audiovisual–motor synchronization, and the repetition of stereotyped movement patterns and vestibular reflexes. The IVE program consists of individualized exercise regimes that offer moderate and strong vestibular stimulus and are rich in invariant cross-modal features.

The at-home protocol included passive and active vestibular exercises. Passive exercises were delivered by the parent(s); active vestibular exercises were performed by the child. Examples of passive vestibular exercises include repeats of rocking motions, horizontal rotations, and vertical side rolls—all in a blanket held by the parents on each side of the blanket—backward dip from the parent’s lap, sit-ups in the parent’s lap (parent holding the child’s hand), and swinging upside-down with the parent holding the child’s hip (parent is sitting) or ankle (parent is standing).

Examples of active vestibular exercises include frontal and backward summersaults, horizontal log rotations (optionally holding balls between hands and/or legs), jumping on a trampoline, rocking back and forth on an exercise ball (both in prone position and sitting), performing handstand from a large exercise ball (diameter: 95 cm), pulling oneself forward and backward in a prone position on a skateboard (legs holding a ball, optionally), pushing oneself backward in a prone position on a skateboard (legs holding a ball, optionally), and pulling back and forth with the legs fixed on the chest of the sitting parent while lying in a supine position (hands optionally holding a ball above the supine chest).

Difficulty was determined according to individual skill levels. Accordingly, the selection of the exercises was determined individually by the physiotherapist at the beginning of each of the 8-week-long regimes; each exercise was to be repeated 10 times per session. Motor performance was always accompanied by some form of language production, e.g., the parent and the child counting, chanting, or singing out loud (preferably together). The length of the daily sessions was around 25 min.

It was hypothesized that individualized vestibular exercises, by delivering strong vestibular stimulation, may activate the parieto-insular vestibular cortex and/or the posterior parietal cortex to contribute to the activation of the cortical areas needed for the maturation of multisensory neurons. Furthermore, exercises were curated to have intersensory redundancy derived from the simultaneous presentation of the same information over several modalities, providing a mechanism for increasing the precision of the multi-modal percept [24]. Children received the same exercises in 8-week-long regimes with 1-week-long training-free intervals interspersed. Overall, three 8-week-long exercise regimes were administered. Each regime started at moderate intensity (5 days of exercise per week for 2 weeks, moderate vestibular stimulation) followed by vigorous intensity (7 days of exercise per week for the period between weeks 3–6, strong vestibular stimulation) and moderate intensity for the last 2 weeks. At the beginning of each treatment regime, parents were trained by the physiotherapist to deliver the exercises properly at home. Adherence was assessed by interviewing the parents on weeks 4, 9, 14, and 26. Adherence was assessed by collecting the parents’ self-report forms, on which they were instructed to track session-to-session progress regarding the execution of exercises. Adherence was defined as a 75% completion rate of exercises in each 8-week-long blocks, high adherence was defined as 90% or higher rate of completion, and low adherence was defined as 75–89% rate of completion.

### 2.2. Statistical Analysis

According to the Shapiro–Wilk test, most of the continuous variables were not normally distributed. Age was transformed as 1/age to obtain normal distribution. Otherwise, when making between-group comparisons (children with DLD vs. the control group) at baseline or at 6-month follow-up, the Mann–Whitney two-sample statistic was used. When testing for equality on matched data (e.g., comparing baseline and follow-up results within the same group), the Wilcoxon matched-pair signed-rank test was performed. Age distribution for the DLD and TD groups was compared by implementing the *t*-test (using 1/age). For these analyses, Stata 13.0 software (Stata Corporation) was used.

To account for basic covariates, linear regression was performed for the primary outcome measure, the composite primitive reflex profile score (squared), and the age-normalized standard points for the TROG-2 test (squared). Transformations were made to achieve normal distribution. Analysis was performed after adjusting for basic covariates, e.g., sex, age at baseline, baseline score (square root and identity for baseline primitive reflex profile score and TROG-2 points, respectively), and history of past rehabilitation efforts. Unadjusted and adjusted results are presented with regression coefficients, 95% confidence intervals (CIs), and *p* values. To assess the presence on an interaction between the group and the dependent variable (composite of primitive reflex profile score and TROG-2 points at baseline), adjusted models were stratified with respect to group allocation (neurotypical children vs. children with DLD).

In addition, to assess the effect of the 26-week-long IVE program on the DLD group, mixed-effects analysis (using REML method) followed by Fisher’s LSD test was performed using GraphPad Prism 10 for Windows (GraphPad Software Inc., La Jolla, CA, USA). The results of this analysis are presented with the understanding that they were obtained from data not following a Gaussian distribution. The goodness of fit was characterized with the REML criterion. Values are expressed as the median (with interquartile range: IQR), and 95% CIs were provided.

## 3. Results

Overall, 44 children were included (mean age 74.75 months (SD ± 9.18 months), 13 females), of whom 20 (aged between 60 and 102 months, mean age 73.1 months (SD ± 12.67 months)) had DLD and 24 (aged between 68 and 87 months, mean age 76.125 months (SD ± 4.55 months)) served as the control population (*p* = 0.28). Gender distribution varied across the two populations, albeit this variation did not reach statistical significance (ten girls vs. three girls in the DLD and control group, respectively, *p* = 0.054). Dropout rate was 10% (two children) in the DLD group (information regarding the reason for dropping out could not be obtained). Every participant completed the examinations at 26-week follow-up in the control group. Data of every participant who completed the follow-up investigation was analyzed. Every child who completed the 26-week-long program was highly compliant (had a 90% or higher adherence rate, Table 1). Clinical characteristics regarding the pregnancy and peripartum period were similar in the two groups, while neurodevelopmental status was significantly poorer in the DLD group. Conversely, higher proportion of children in the DLD group received some form of rehabilitation (e.g., cognitive or somatosensory) earlier (Table 2).

### 3.1. Baseline Comparisons

Primitive survival reflexes showed limited integration among children suffering from developmental language disorder. The score for ATNR was significantly lower (0 IQR 0–0.5 vs. 2 IQR 2–2) for the DLD and control groups, respectively; (*p* < 0.001), similarly to that of STNR (0 IQR 0–1 vs. 2 IQR 1–2 for the DLD and control groups, respectively; *p* < 0.001). The Schilder’s test was conversely worse in the DLD group (0.5 IQR 0–1.5 vs. 2 IQR 1–2 for the DLD and control groups, respectively; *p* = 0.017). Tonic labyrinth reflex scores were also significantly lower among children with DLD (2 IQR 1–4 vs. 6 IQR 5–6 for the DLD and control groups, respectively; *p* < 0.001). Regarding the score for co-contraction, reflective of dynamic postural control, the results were also similar (0.25 IQR 0–1 vs. 2 IQR 2–2 for the DLD and control groups, respectively; *p* < 0.001). Furthermore, age-standardized scores for receptive grammar were significantly lower in the DLD group (79.5 IQR 71.5–89.5 vs. 103 IQR 97–109 for the DLD and control groups, respectively; *p* < 0.001).

### 3.2. Results at Follow-Up

Compared to baseline values, the primitive reflex profile improved in the DLD group. The score for ATNR increased to 2 (IQR 1–2) (*p* < 0.001 for baseline vs. follow-up) and for the Schilder’s test it increased to 1 (IQR 1–2) (*p* = 0.018 for baseline vs. follow-up). Similarly, the presence of STNR also diminished, with the score for STNR being 2 (IQR 2–2) (*p* < 0.001 for baseline vs. follow-up). The tonic labyrinth reflex profile also improved when compared to baseline (median score at follow-up 5 IQR 4–6, change vs. baseline *p* = 0.001). Furthermore, dynamic postural control (score of 2 IQR 1–2) (*p* < 0.001 for baseline vs. follow-up), as well as age-standardized scores for receptive grammar, improved significantly (score of 87 IQR 66–103; *p* = 0.03 for baseline vs. follow-up).

As for the control group, the only significant difference between the baseline and follow-up results concerned the Schilder’s test, which showed an improvement (score 2 IQR 1.5–2; *p* = 0.045 for baseline vs. follow-up).

**Figure 1:**
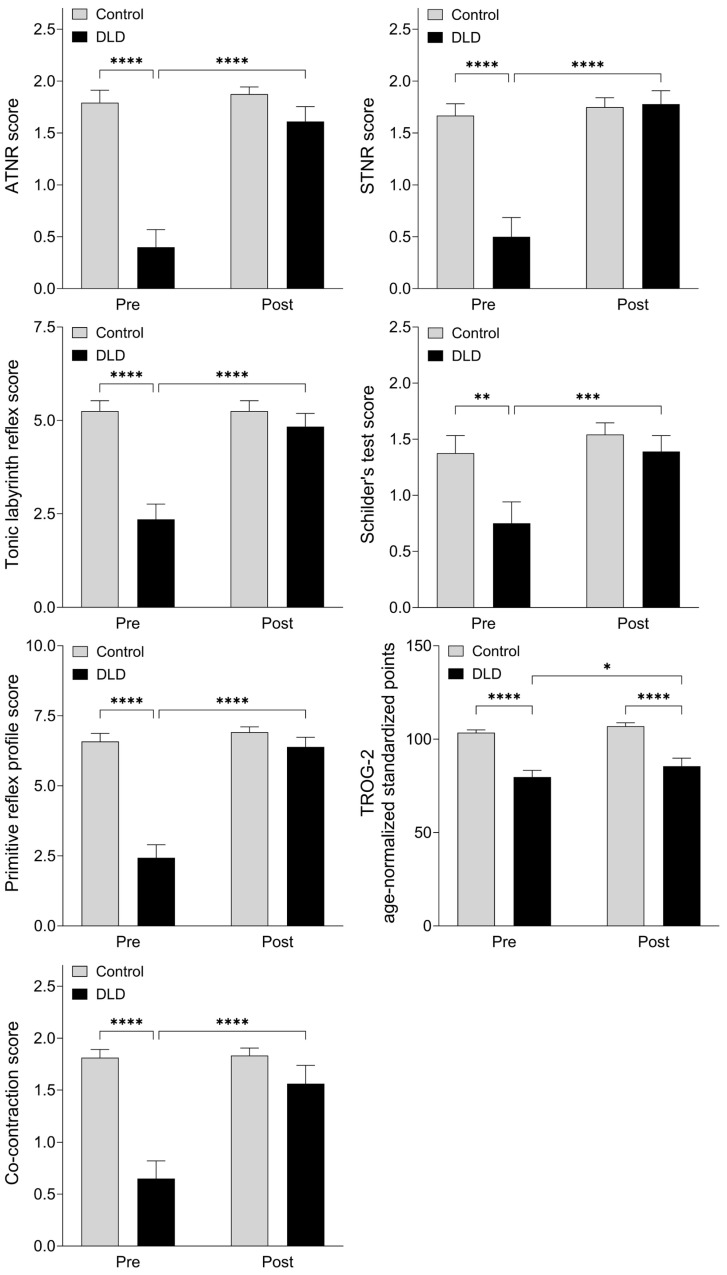
**Primitive reflexes, postural control, and receptive language performance** in children with developmental language disorder (DLD) and controls showing typical neurodevelopment at baseline (pre) and at 26-week follow-up (post). ATNR, STNR, and Schilder’s test are scored on a scale of 0–2; the assessment of TLR includes three subdomains, each evaluated on a scale of 0–2 points, the sum of which are illustrated. The primitive reflex profile is characterized as the sum of scores for ATNR, STNR, Schilder’s test, and the arithmetic mean of TLR subdomains. Receptive language performance is characterized by the age-normalized standardized points on the TROG-2 test. Dynamic postural control is characterized by the co-contraction scores. *: *p* < 0.05; **: *p* < 0.01; ***: *p* < 0.001; ****: *p* < 0.0001. TLR (tonic labyrinth reflex), ATNR (asymmetric tonic neck reflex) and STNR (symmetric tonic neck reflex).

**Figure 2:**
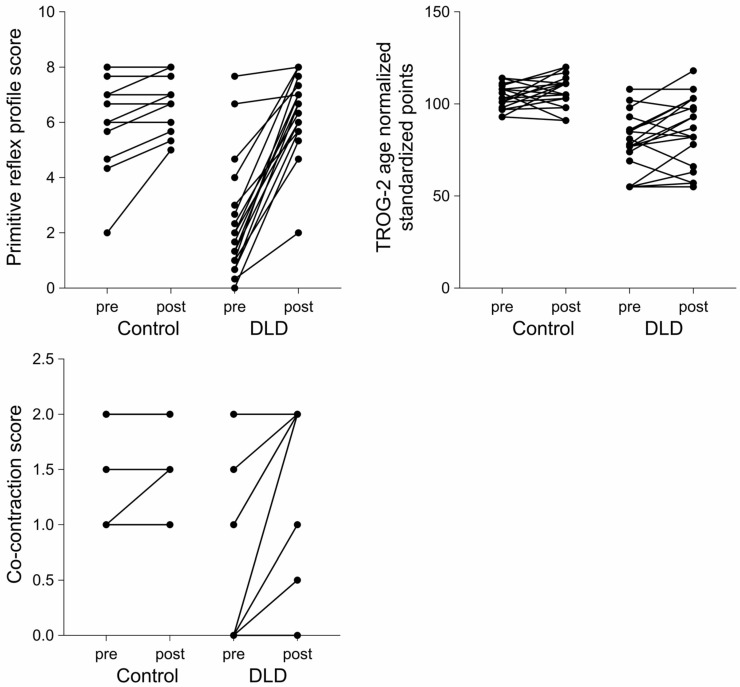
**Individual trajectories of the composite score for primitive** reflexes, postural control, and receptive language performance in children with developmental language disorder (DLD) and controls showing typical neurodevelopment at baseline (pre) and at 26-week follow-up (post). The primitive reflex profile is characterized as the sum of scores for ATNR, STNR, Schilder’s test, and the arithmetic mean of TLR subdomains. Receptive language performance is characterized by the age-normalized standardized points on the TROG-2 test. Dynamic postural control is characterized by the co-contraction scores.

When two-way comparisons using the mixed-effects model were made, an improvement in the DLD group was evident in comparison with the group of children showing neurotypical development. There was no significant difference with respect to the primitive reflex profile characterized by ATNR, STNR, TLR, Schilder’s test, and the summary primitive reflex profile score when the results of the control groups were compared at baseline and follow-up. The case was considerably different in the DLD group, where the scores reflecting primitive reflex profile were significantly higher at follow-up. The baselines between group comparisons were significantly different between the DLD and control group, a difference that was no longer present at follow-up, indicating that the primitive reflex profile in the DLD improved and became similar to that of the control group. The results were similar regarding dynamic postural control (Figure 1, Table 3). At baseline, the scores for co-contraction were significantly lower in the DLD group than in the control group, a difference that diminished at 26-week follow-up (Figure 1, Table 3). Age-normalized standardized points were significantly higher in the control group both at baseline and at follow-up, with significant improvement seen at 26 weeks in the DLD group (Figure 1, Table 3). Mixed-effects analysis (with Fisher’s LSD test) yielded similar results, with evidence of the interaction between the groups (DLD vs. control group) and time (baseline vs. follow-up) being significant in all domains except for the TROG-2 test scores (Table 4). Individual trajectories are illustrated in Figure 2.

### 3.3. Linear Regression Analysis

Linear regression analysis showed a significant relationship between the composite of primitive reflex profile score at baseline and at follow-up (β = 10.87 95%; CI: 6.11–15.64; *p* < 0.001). This relationship remained significant after adjustment for covariates (Table 5, panel A). Similarly, the age-normalized standardized points for the TROG-2 test at baseline and follow-up were significantly correlated (β = 146.00; 95% CI: 109.50–182.50; *p* < 0.001), an effect that also remained significant after adjusting for covariates (Table 5, panel B). Stratification by group, altered regression coefficients for both outcome measures assessed. The regression coefficient of the composite primitive reflex profile score at baseline was 37.26 (95% CI: 24.98–49.53; *p* < 0.001) and 13.52 (95% CI: 2.47–24.57; *p* = 0.021) for the neurotypical and DLD group, respectively. The regression coefficient of the TROG-2 scores at baseline also changed to 71.08 (95% CI: −71.60–213.77; *p* = 0.31) and 100.69 (95% CI: 0.39–200.98; *p* = 0.049), respectively, for the neurotypical and DLD groups after stratification.

## 4. Discussion

The current study shows an improvement in the integration of primitive survival reflexes, receptive language skills, and postural control among children with DLD who underwent the 26-week-long IVE program, providing intense vestibular stimuli. Primitive reflex profile and postural control improved to the extent that there was a lack of statistically significant difference with the control group at follow-up. Receptive language performance also improved significantly at follow-up, albeit there remained a significant difference between the two groups. As the group × time interaction for the normalized standardized points for the TROG-2 test was statistically non-significant, it cannot be ruled out that the observed improvement in language scores may reflect natural developmental progress rather than a treatment effect.

Starting from the finding that both postural control [39] and receptive language development [5] are rooted in multisensory and sensorimotor integration, we propose that, in children with DLD, the capability for multisensory and sensorimotor integration increased. The possible role of intensive vestibular exercise program may be proposed, however, due to the study design, cause and effect cannot be established at this time. Similarly, it can neither be concluded whether an improvement in postural control and receptive language performance is linked to the integration of primitive reflexes. Nonetheless, the integration of primitive reflexes coincided with a marked improvement in two distinct processes involving MSI and SMI. Whether this improvement in primitive reflex profile reaches clinical significance could be amenable to debate, albeit there are some prior publications that report on the beneficial effects of integration of a single primitive reflex (e.g., see ATNR or STNR, TLR, etc. [40]). Additionally, these improvements emerged after a 26-week-long intensive vestibular treatment regime. These findings tempt one to speculate that the possible common denominator for change is vestibular stimulation. Others have reported results that corroborate the possible role of the vestibular system. A cross-sectional study assessed the extent of co-occurrence between persisting primitive reflexes, balance deficits, and symptoms of attention deficit/hyperactivity disorder (ADHD) in a group of children with ADHD and their TD peers. The authors found a strong association between cognitive and motor dysfunction, disturbed balance, and persisting primitive reflexes, and proposed that movement intervention programs targeting the integration of primitive reflexes may be useful in clinical or classroom settings to improve cognitive performance [41].

MSI and SMI are based on the ability to decode the hidden regularities of the environment. This process is fundamental from birth, as continuous acquisition of sensory experiences will be used to develop a world model using Bayesian inference. Accordingly, sensory representations of multisensory areas, e.g., the superior colliculi and the posterior parietal cortex, are topographically organized in a way that the multiple excitatory receptive fields for each modality they respond to show spatial correspondence [42]; therefore, cross-modal activation with respect to a single event will repeatedly activate the same multisensory neuron through separate sensory systems. Under the premises of MSI, if distinct unimodal stimuli fall on overlapping excitatory receptive fields, enhancement superseding the sum of individual neuronal responses may develop. Conversely, subadditivity ensues if one modality falls outside this excitatory zone. These early experiences cause spatially overlapping receptive fields to be sculpted from their larger neonatal templates during development [7]. For example, if the spatial distance between unisensory inputs increases to the extent that activation from one modality falls on the inhibitory border zone of another modality, neuronal response is depressed and performance is diminished [42]. Retained primitive reflexes may hinder the development of MSI and SMI, as they interfere with proper decoding of hidden environmental regularities. This, by impeding the ability to deduce the statistical regularities underlying co-occurring events, will prolong the accumulation of experiences required to draw statistical inference, so the development of processes reliant on MSI and SMI (for example, postural stability and language development) will show a protracted course.

Experimental evidence regarding how receptive fields cancel out different signals was presented by Brooks and colleagues. In adult monkeys, they have shown that intact vestibulospinal reflexes and proprioceptive and vestibular signals cancel each other out when the head is passively moved relative to a stationary body [43], a maneuver known to elicit ATNR in infancy [44]. Further empirical evidence is provided if one considers the observation related to the natural course of reflex integration, e.g., repetition of stereotyped movements, such as crawling or rocking on hands and knees, leads to the integration of ATNR and STNR, respectively [45]. Moreover, there is evidence that the standardized repetition of stereotyped infant movements inhibits ATNR [46]. How repetition may factor into reflex inhibition is not yet known, but it may be hypothesized that variations during repetitive movements accidentally lead to the activation of inhibitory sidebands, thus occasionally reducing the response and thereby, on average, reducing the effect caused; hence, repetitive activation of the inhibitory marginal zones may shape the receptive field to develop the inhibitory subregions, reducing the response of the multisensory units that corresponded with the primitive reflex. Thus, coincident sensory cues will fall on either the inhibitory marginal zone of the previously joined receptive fields or outside of it, dividing the cross-modal attributes of primitive reflexes. This may possibly contribute to the degradation of primitive reflexes. Exercise programs targeting the repetition of the stereotypical neonatal movements associated with primitive reflex patterns have been proposed to result in reflex integration, an effect that was parallelled by improved reading, writing, and spelling performance, as well as phonological skills [46]. Previously, it was proposed that rhythmic movement activities, balance, and whole-body coordination exercises facilitate the integration of primitive reflexes and, in fact, may mediate beneficial neurodevelopmental effects [32, 41].

To the best of our knowledge, this is the first study which assesses the effect of vestibular stimulation on the processes that rely on multisensory and sensorimotor integration (e.g., postural control, primitive reflex integration and receptive language performance) in children with DLD. Evidence underscoring the theoretical proposal that vestibular stimulation is beneficial for improving multisensory and sensorimotor integration comes from studies conducted on populations distinct from ours. In an interventional study involving thirty-three healthy volunteers, the effect of no training, postural training, and postural training coupled with active horizontal headshakes that can induce vestibular perturbations was assessed. Rhythmic horizontal headshake training increased postural stability, possibly by sensorimotor processes that lead to sensory reweighting and lower-level adaptation of visual–vestibular processes [39]. Further evidence for multisensory integration was provided in an EEG study. Forty-two healthy adults were instructed to keep their balance on a balance board while virtual reality stimuli created multisensory conflict among different sensory modalities. The authors found that postural stability improved when only congruent sensory modalities were utilized in sensory integration and the incongruent visual input was omitted [47]. The beneficial effect of vibrotactile sensory augmentation regarding postural stability is another piece of supporting evidence for the interplay between multisensory integration, vestibular stimulation, and postural control. With the involvement of 16 participants, the effect of eight-week in-home balance training with or without vibrotactile sensory augmentation was assessed on postural sway. An improvement was seen in both groups; however, a minimal detectable change of 8 points in the Sensory Organization Test scores was only present in the experimental group. Additionally, four participants from the experimental group underwent fMRI examination, which showed a change in the pattern of brain activity. Following training, the brainstem and cerebellar regions were more involved. The authors suggested that sensory augmentation alters the neural processing of vestibular stimulation by means of sensory reweighting mechanisms switching from cortical to subcortical involvement [48].

More compelling evidence for the positive effect of structured exercises targeting rhythm, balance, and coordination was recently provided by assessing children with neurodevelopmental disorder. Overall, 12 children with ADHD and 15 children with autism spectrum disorder underwent a 12-week long program and, at follow-up, persistent primitive reflexes were found to be significantly reduced (especially ATNR), parallelled by behavioral improvement and improved attention. The authors proposed that, among others, sensorimotor integration may mediate the observed changes [32].

The interplay between sensory profile and primitive reflexes was also assessed previously. In a cross-sectional study of 44 preschool children, the presence of nonintegrated reflexes was most strongly correlated with disturbed sensory processing such as dyspraxia, sensory–vestibular, and postural disorders [49]. Another study evaluated the prevalence of primitive reflexes in children with developmental language disorder and age-matched typically developed children. The co-occurrence of nonintegrated reflexes and poorer performer on the nonword repetition test, a sensitive marker for developmental language disorder, was identified. The authors believed that uninhibited primitive reflexes can be important in the context of developmental language disorder [31]. Similar findings were reported regarding the efficacy of rhythmic movement training [50], a treatment program designed to aid the integration of primitive reflexes. The integration of retained primitive reflexes paralleled by improvement in cognitive, physical, and social skills was reported by families implementing this home-based training program [51]. Others have reported the beneficial effects of a 12-week rhythmic, balance, and whole-body coordination exercise program to enhance reflex integration, parallelled by improved behavioral and motor development in children with autism spectrum disorder and attention deficit/hyperactivity disorder [32].

The current study has some limitations. The intervention group had considerably more boys than the control group (albeit this difference was not statistically significant). Nevertheless, selection bias was addressed by inviting every eligible child to participate in the study. Blinding the investigator was not possible, as every child with DLD was included in the interventional study, and the language impairment became evident when interacting with the children (interaction was necessary to ensure compliance with the examination and the exercises). Another debatable shortcoming is that the control group did not undertake IVE training during the follow-up period. The reason for this is that the IVE program is designed to be administered if certain clinical indications are present. Completion of the training program for 6 months mandates great effort from both the parents and the child. Due to the lack of clinical indication for IVE training and its toll on the participants, the control group received no intervention during follow-up. Nonetheless, it must be acknowledged that this design limits the strength of our conclusions and does not rule out nonspecific effects (e.g., attention, repeated testing, etc.). Furthermore, there was a lack of engagement on behalf of the parents of the control group to regularly deliver a general exercise program (three times a week) during the follow-up period. Due to this, the general effect of exercise remained uncontrolled for. However, follow-up data from the control group was included in the analysis because it may add to the interpretation of our results, as it makes it possible to account for the effect of spontaneous neurological development when making four-way comparisons (intervention vs. no intervention, baseline vs. follow-up).

Our study has some merits as well. The children participating in the IVE group underwent a relatively long training regime, supervised by the same physiotherapist. This eliminated the variability coming from the supervisor. Furthermore, the IVE program was individually tailored to the specific areas showing developmental delays (e.g., the extent of retained primitive reflexes, delays in gross motor and fine motor function, etc.). Additionally, the current study carries some implications for speech–language therapy and physiotherapy, calling attention to the need to broaden the cross-disciplinary nature of DLD interventions. Physiotherapists should be advised to include speech–language therapists in formal assessment for language development when working with children who have retained primitive reflexes or postural instability and vice versa. Children at risk of, or identified with, DLD should be assessed by a physiotherapist with respect to postural control and retained primitive reflexes.

The current findings, e.g., the improvement in primitive reflex profile and postural control and receptive language, processes which are reliant on MSI and SMI, may have implications for understanding the etiopathogenetic processes contributing to DLD. The interplay between MSI and SMI, primitive reflex integration, and language, driven by vestibular stimuli, could be further evaluated in epidemiological studies and controlled clinical trials. Prospective cohort studies may allow for the parallel assessment of motor development and multisensory and sensorimotor integration over a longer three- to five-year-long period. Furthermore, controlled clinical trials may be implemented to quantify the beneficial effects that reflex integration therapies, occupational therapy, and sensory integration therapy may have on primitive reflex inhibition and motor development paralleled by multisensory and sensorimotor integration. These studies may be further augmented by fMRI studies that may characterize the change in functional connectivity in motor and default mode networks, previously shown to be related to changes in network-level brain–behavior relationships paralleling gross motor development [52]. It is worthwhile of notice that these studies must be structured that so differences stemming from the natural variations in typical development are accounted for. Although the results are promising, it is not currently possible to imply a direct relationship between the integration of primitive reflexes and improvements in language.

## 5. Conclusions

We hypothesized that multisensory and sensorimotor integration is needed for the integration of primitive reflexes and that vestibular stimuli may be the potential cortical input that contributes to the maturation of multisensory areas underlying these processes. It would further follow that perturbations of multisensory integration may contribute to the persistence of primitive survival reflexes that often accompany developmental delays. The current pilot interventional study demonstrated the potential benefits of vestibular stimulation in children with DLD. That the supervised training program vested in vestibular stimuli contributed to improvements in primitive reflex profile and multisensory processes possibly offers some implicit clinical evidence in support of our hypothesis.

## Figures and Tables

**Table 1:** Adherence to the exercise regime in the DLD group over the three consecutive 8-week-long exercise regimes.

Block	Block 1								Sum (%)	Block 2								Sum (%)	Block 3								Sum (%)
Week	1	2	3	4	5	6	7	8		1	2	3	4	5	6	7	8		1	2	3	4	5	6	7	8	
Exercise Sessions per Week	5	5	7	7	7	7	5	5		5	5	7	7	7	7	5	5		5	5	7	7	7	7	5	5	
**ID 1**	5	5	7	7	7	7	5	5	48 (100%)	5	5	7	7	7	7	5	5	48 (100%)	5	5	7	7	7	7	5	5	48 (100%)
**ID 2**	5	5	7	7	7	7	5	5	48 (100%)	5	5	7	7	7	7	5	5	48 (100%)	5	5	7	7	7	7	5	5	48 (100%)
**ID 3**	5	5	7	7	7	7	5	5	48 (100%)	5	5	7	7	7	7	5	5	48 (100%)	5	5	7	7	7	7	5	5	48 (100%)
**ID 4**	4	5	7	7	7	7	5	5	47 (98%)	1	5	7	7	7	7	5	5	44 (91%)	5	5	7	7	7	7	5	2	45 (93%)
**ID 5**	5	5	7	7	7	7	5	5	48 (100%)	5	5	7	7	3	7	5	5	44 (91%)	5	5	7	7	7	7	5	5	48 (100%)
**ID 6**	4	4	7	7	7	7	5	5	46 (95%)	5	5	7	7	7	7	2	4	44 (91%)	4	3	6	7	7	7	5	5	44 (91%)
**ID 7**	5	5	7	7	7	7	5	5	48 (100%)	4	5	7	7	7	7	5	5	47 (98%)	5	5	7	7	7	7	5	5	48 (100%)
**ID 8**	5	5	7	7	7	7	5	5	48 (100%)	4	5	7	7	7	7	5	5	47 (98%)	4	4	6	7	7	7	5	5	45 (93%)
**ID 9**	5	5	7	7	7	7	5	5	48 (100%)	5	5	6	7	7	7	5	5	47 (98%)	5	5	7	7	7	7	5	5	48 (100%)
**ID 10**	5	5	7	7	7	7	5	5	48 (100%)	5	5	7	6	7	7	5	5	47 (98%)	5	5	7	7	7	7	5	5	48 (100%)
**ID 11**	5	5	7	7	7	7	5	5	48 (100%)	4	5	7	7	7	7	5	5	47 (98%)	5	5	7	6	7	7	5	5	47 (98%)
**ID 12**	4	4	6	7	7	6	5	5	44 (91%)	5	5	6	7	7	7	5	5	47 (98%)	5	5	6	7	7	7	5	5	47 (98%)
**ID 13**	5	5	7	7	7	7	5	5	48 (100%)	4	5	7	7	7	7	5	5	47 (98%)	5	5	7	7	6	7	5	5	47 (98%)
**ID 14**	5	5	7	7	7	7	5	5	48 (100%)	5	5	7	7	7	7	5	4	47 (98%)	5	5	7	7	7	6	5	5	47 (98%)
**ID 15**	5	5	7	7	7	7	5	3	46 (95%)	5	5	7	7	7	7	5	4	47 (98%)	5	5	7	7	7	7	4	5	47 (98%)
**ID 16**	5	5	4	7	7	7	5	5	45 (93%)	5	5	6	7	7	7	5	5	47 (98%)	5	5	7	7	7	7	5	4	47 (98%)
**ID 17**	3	5	7	7	7	7	5	5	46 (95%)	5	5	7	7	7	7	5	5	48 (100%)	5	5	7	7	7	6	5	5	47 (98%)
**ID 18**	5	5	7	5	7	7	5	5	46 (95%)	5	5	7	7	7	7	5	5	48 (100%)	5	5	7	7	6	7	5	5	47 (98%)
**ID 19**	5	5	7	7	7	7	5	5	48 (100%)	5	5	7	7	7	7	5	5	48 (100%)	5	5	7	6	7	7	5	5	47 (98%)
**ID 20**	5	5	7	7	7	7	5	5	48 (100%)	5	5	7	7	7	7	5	5	48 (100%)	5	5	7	7	7	7	5	2	45 (93%)

**Table 2:** Baseline clinical characteristics of the DLD and TD groups. Data is presented as median (interquartile range), unless otherwise specified.

Characteristic	TD Group	DLD Group	*p*
***Characteristics related to the pregnancy***			
N° of pregnancies	2 (1–3)	2 (1–2)	0.251
Complicated pregnancy according to the physician (n/y)	14/5	10/10	0.129
Gestational diabetes (n/y)	17/2	19/1	0.517
Gestational hypertension (n/y)	18/1	18/2	0.579
Toxemia (n/y)	19/0	20/0	-
Problems with placenta (n/y)	17/2	19/1	0.517
Problems with amniotic fluid (n/y)	16/3	17/3	0.946
***Birth related characteristics***			
Time of birth (gestational week)	39 (38, 40)	39 (34, 40)	0.743
Birth weight (g)	3230 (3050, 3850)	3210 (2350, 3710)	0.346
Birth length (cm)	50 (49, 52)	50 (47, 52)	0.80
Birth head circumference	34 (32.5, 35)	34 (30, 34)	0.20
Cesarean section (n/y)	9/10	9/11	0.556
Perinatal infection (n/y)	17/2	19/1	0.517
Perinatal hypoxia (n/y)	18/0	18/2	0.168
Perinatal skull trauma (n/y)	19/0	19/1	0.323
Perinatal bone fracture (n/y)	19/0	19/1	0.323
***Neurological function***			
CNS maturity (points)	24 (22, 26)	8.5 (6, 13.5)	<0.001
Gross motor performance (points)	16 (13.5, 16)	2 (0, 10)	<0.001
Body schema (points)	37.5 (34, 40)	10.5 (5, 15.5)	<0.001
Tactile perception (points)	49 (46.5, 54)	5 (0, 27.5)	<0.001
Rhythmic performance (points)	6 (2, 10)	0 (0, 2.5)	<0.001
***Prior rehabilitation***			
Cognitive rehabilitation (n/y)	19/0	9/9	<0.001
Sensorimotor rehabilitation (n/y)	17/2	6/12	<0.001
Physiotherapy rehabilitation (n/y)	18/1	15/3	0.264

**Table 3:** Medians, interquartile range and 95% confidence interval (CI) for the primary outcome measure composite of primitive reflex profile score (Prim) and its components (ATNR, STNR, TLR, Shilder test), the age-standardized normalized points for the TROG-2 test (TROG), and the measure of dynamic postural control (Co-con) at baseline (pre) and at 26-week follow-up (post) in the two groups (neurotypical children—control group and children with DLD). Legend: TLR (tonic labyrinth reflex), ATNR (asymmetric tonic neck reflex), STNR (symmetric tonic neck reflex), and Schild (Schilder test).

	**Control**													
	**ATN Pre**	**ATNR Post**	**STNR Pre**	**STNR Post**	**TLR Pre**	**TLR Post**	**Schild Pre**	**Schild Post**	**Prim Pre**	**Prim Post**	**TROG Pre**	**TROG Post**	**Co-Con Pre**	**Co-Con Post**
**Median**	2	2	2	2	6	6	2	2	6.8	7	103	111	2	2
**25; 75 p.**	2; 2	2; 2	1; 2	1.25; 2	5; 6	5; 6	1; 2	1; 2	6; 7.9	6.2; 8	97; 109.5	100.5; 111	2; 2	2; 2
**95% CI**	2; 2	2; 2	1; 2	2; 2	5; 6	5; 6	1; 2	1; 2	6; 7.7	6.7; 8	97; 108	103; 111	2; 2	2; 2
	**DLD**													
	**ATN Pre**	**ATNR Post**	**STNR Pre**	**STNR Post**	**TLR Pre**	**TLR Post**	**Schild Pre**	**Schild Post**	**Prim Pre**	**Prim Post**	**TROG Pre**	**TROG Post**	**Co-con Pre**	**Co-con Post**
**Median**	0	2	0	2	2	5	0.5	1	1.8	6.7	79.5	87	0.25	2
**25; 75 p.**	0; 0.8	1; 2	0; 1	2; 2	1; 4	4; 6	0; 1.8	1; 2	1; 3.8	5.7; 7.4	70.3; 91.3	66; 103	0; 1	1; 2
**95% CI**	0; 0	1; 2	0; 1	2; 2	1; 4	4; 6	0; 1	1; 2	1; 3	5.7; 7.3	74; 86	66; 103	0; 1	1; 2

**Table 4:** The effect of IVE training on children in the DLD group (obtained with mixed-effects analysis). The goodness of fit was characterized with the REML criterion. IVE: individualized vestibular exercise; ATNR: asymmetric tonic neck reflex score; STNR: asymmetric tonic neck reflex score; TLR: tonic labyrinth reflex score; Schilder: Schilder test score; Prim. sum: summary primitive reflex profile score; TROG-2: Test for Reception of Grammar-2 score; Co-contr.: co-contraction score; Pre: baseline; Post: follow-up; Co: control group; DLD: group of children diagnosed with developmental language disorder; TLR (tonic labyrinth reflex), ATNR (asymmetric tonic neck reflex) and STNR (symmetric tonic neck reflex); *: *p* < 0.05; **: *p* < 0.01; ***: *p* < 0.001; ****: *p* < 0.0001. ns: statistically non-significant.

	ATNR	STNR	TLR	Schilder	Prim. Sum	TROG-2	Co-Contr.
**Time** (Pre to Post)	<0.0001	<0.0001	<0.0001	0.0004	<0.0001	0.006	<0.0001
	****	****	****	***	****	**	****
**Group** (Co and DLD)	<0.0001	0.0014	0.0002	0.0344	<0.0001	<0.0001	0.0002
	****	**	***	*	****	****	***
**Interaction** (Time x Group)	<0.0001	<0.0001	<0.0001	0.0361	<0.0001	0.3958	<0.0001
	****	****	****	*	****	ns	****
**REML criterion**	73.8	78.3	152.4	86.9	148.7	317.7	66.2
**Pre:** Co vs. DLD	<0.0001	<0.0001	<0.0001	0.0039	<0.0001	<0.0001	<0.0001
	****	****	****	**	****	****	****
**Post:** Co vs. DLD	0.2174	0.7644	0.3865	0.3496	0.2814	<0.0001	0.407
	ns	ns	ns	ns	ns	****	ns
**Co:** Pre vs. Post	0.4572	0.4979	>0.9999	0.2017	0.2188	0.1437	0.827
	ns	ns	ns	ns	ns	ns	ns
**DLD:** Pre vs. Post	<0.0001	<0.0001	<0.0001	0.0003	<0.0001	0.0135	<0.0001
	****	****	****	***	****	*	****

**Table 5:** The primary and secondary outcome measures adjusted for basic covariates by means of linear regression. Panel A shows the regressors for the composite primitive reflex profile scores at follow-up. Panel B shows the regressors for the age-normalized standardized points for TROG-2 test at follow-up (*p*, level of significance) (note that the outcome measure and some regression parameters were transformed; see Section 2).

Panel A	Regression Coefficient	95% Confidence Interval	*p*
baseline composite points for primitive reflexes (square root)	18.48	11.56; 25.40	<0.001
age (in months)	0.051	−0.42; 0.52	0.83
sex (female 0, male 1)	1.23	−6.98; 9.43	0.76
group (neurotypical 0, DLD 1)	14.00	1.03; 26.99	0.035
past rehabilitation (no 0, yes 1)	1.04	−4.79; 6.86	0.72
constant	−3.15	−44.58; 38.29	0.88
**Panel B**	**Regression** **coefficient**	**95% confidence interval**	***p***
baseline TROG-2 point	118.94	54.01; 183.87	0.001
age (in months)	−54.76	−143.12; 33.60	0.22
sex (female 0, male 1)	−912.38	−2345.54; 520.78	0.20
group (neurotypical 0, DLD 1)	−1741.68	−3919.30; 435.30	0.11
past rehabilitation (no 0, yes 1)	620.40	−399.83; 1640.64	0.22
constant	3826.87	−7733.20; 15,386.92	0.50

**Figure A1:**
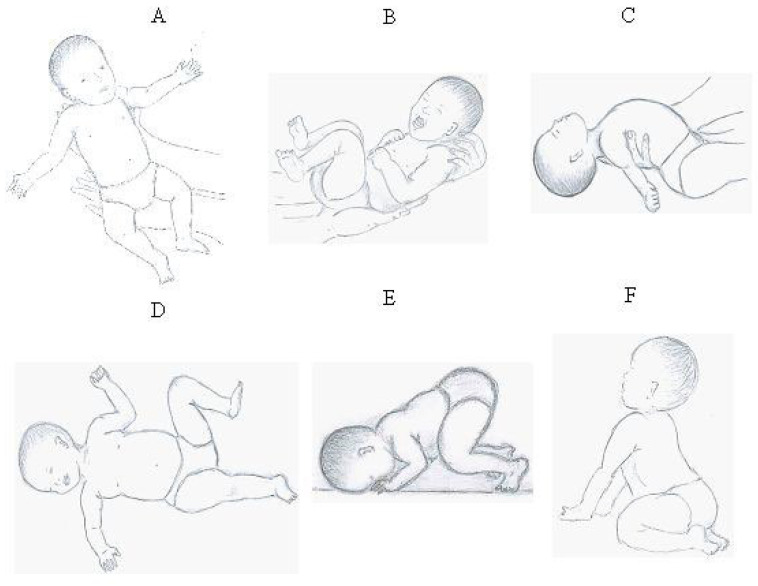
Illustration of primitive survival reflexes ((**A**): Moro reflex; (**B**): prone tonic labyrinth reflex; (**C**): supine tonic labyrinth reflex; (**D**): asymmetric tonic neck reflex; (**E**): symmetric tonic neck reflex–anteflexion of the head; (**F**): symmetric tonic neck reflex–retroflexion of the head).

## Data Availability

Regarding data and materials used for this work, any inquiries can be directed to the corresponding author.

## Appendix A

Of the numerous primitive survival reflexes, the Moro reflex, the TLR, the ATNR, and the STNR share several attributes in terms of their mechanisms and influence on central nervous system maturation [26, 28]. These reflexes are all evoked by vestibular stimulation and produce motor output utilizing vestibulo–spinal, vestibulo–cerebellar, and vestibulo–ocular loops [30]. Thus, they are instrumental in developing posture, balance, and coordination, as well as the perceptual processing underlying receptive language, reading, and writing skill development [23, 28].

The Moro reflex develops between the ninth to the twelfth gestational week and normally becomes inhibited by the second to the fourth postnatal month. It may be elicited by sudden loud noises, bright light, change in temperature, or backward drop of the head [25]. As a response, the hands become extended and the infant grasps and then crosses the arms in front of the trunk. Inhibition of Moro reflex occurs at the time that rolling emerges [25]. A retained Moro reflex can contribute to hypersensitivity to light, sound, and temperature, which leads to sympathetic overstimulation and heightened arousal, hence providing a continuous backdrop of angst. On the one hand, this leads to impaired pupillary response to light and poor balance and coordination; on the other, it exhausts the cognitive load, thus hindering the ability to categorize and form perceptual schemas [28, 29, 44].

The tonic labyrinth reflex (TLR), closely bound to the Moro reflex, is fundamental to develop the proper alignment of the head and body with respect to its environment. Prone TLR develops in utero and becomes inhibited around the fourth postnatal month [27]. Anteflexion of the infant’s head causes flexion of all extremities in front of the body. Supine tonic labyrinth reflex develops at birth to become gradually inhibited from 6 weeks to 3 years of age. It may be evoked by retroflexion of the head, causing the extension of the trunk and the extremities. It offers a rudimentary mechanism to cope with gravity. TLR influences the balance between extensor and flexor muscles and, as a result, extensor tone develops in a rostro–caudal direction. This will enable the position of the head to be stabilized against gravity, when it emerges as the reference point for assessing space, distance, and velocity [29, 30]. If retained, the TLR interferes with the development of postural reflexes, especially the ocular and the head-righting reaction. The resultant reciprocal disturbance between the vestibular and visual system will impair oculomotor development and balance, rendering the idiothetic reference frame improper and hindering spatial judgment [28]. Furthermore, lack of sufficient head control will hinder the development of eye–hand control, accommodation, and convergence, all mandatory for visual acuity and coordination [28, 29].

Asymmetric tonic neck reflex (ATNR) emerges at the 18th gestational week and becomes integrated between the third and ninth months. Turning the head to the right side causes the extension of the right arm and leg, paralleled by the flexion of the extremities on the opposite side and vice versa. ATNR splits movements of the two sides of the body vertically. Its inhibition is mandatory to allow for coordination of the two sides as the body prepares for interweaving movement patterns, as seen with creeping, walking, and running [44]. It is instrumental for visual development, as, similarly to TLR, it contributes to eye–hand control. Failure of its inhibition interferes with left–right integration [27] and development of dominance regarding the hands, feet, and eyes [28]. Furthermore, it hinders the eyes’ ability to cross the vertical midline, a skill mandatory for reading and writing, with the latter skill further burdened by poor eye–hand control [27, 30]. Retained ATNR interferes with convergence as symmetrical release of eyes is impaired upon focusing from near to far [44] and leads to poor eye-tracking [27]. Symmetric tonic neck reflex (STNR) is a reflex that emerges parallel to the inhibition of TLR [29]. Some theorize that it is the modification of the TLR seen during its inhibition, as response to the extension of the head will lead to the partial modification of TLR by flexing instead of extending the lower extremities [29]. It surfaces after the ATNR is inhibited; hence, by this stage, the body can coordinate its two sides across the vertical midline, and the eyes can focus on a distant target [30, 44].

STNR emerges postnatally between the age of 6–9 months to become inhibited between 9–11 months of age. If elicited in the quadrupedal position by anteflexion of the head, flexion of the arms and extension of the legs ensues, while retroflexion of the head leads to extension of the arms and flexion of the legs [27, 53]. The STNR will enable the body to move against gravity, facilitates focusing to near distance and for the first time permits the body to lift. This promotes integration of vestibular, visual, and proprioceptive input during movement [44]. Integration of STNR is instrumental to coordinate movements along the horizontal midline, therefore STNR has major contribution to posture and coordination [28, 30]. Delayed STNR integration contributes to predominant flexor tone, leading to poor posture, especially evident when sitting [30]. Furthermore, adjustment of visual focus between different distances will be impaired, leading to poor hand–eye coordination, manifesting in clumsiness and impaired ability to copy graphical elements [28]. Although STNR lifts the body, movement is not yet possible; therefore, its delayed inhibition will impair cross-pattern creeping [29]. Primitive survival reflexes are illustrated in Figure A1.
